# Effects of Jump-Rope-Specific Footwear Selection on Lower Extremity Biomechanics

**DOI:** 10.3390/bioengineering9040135

**Published:** 2022-03-24

**Authors:** Hai-Bin Yu, Jing Li, Rui Zhang, Wei-Ya Hao, Jian-Zhi Lin, Wei-Hsun Tai

**Affiliations:** 1 School of Physical Education, Quanzhou Normal University, Quanzhou 362000, China; yhb@qztc.edu.cn (H.-B.Y.); zhangrui@jlu.edu.cn (R.Z.); haoweiya@ciss.cn (W.-Y.H.); 2 College of Textiles and Apparel, Quanzhou Normal University, Quanzhou 362000, China; lijing2021@qztc.edu.cn; 3 Key Laboratory of Bionic Engineering (Ministry of Education, China), Jilin University, Changchun 130022, China; 4 China Institute of Sport Science, General Administration of Sport of China, Beijing 100061, China; 5 Department of Physical Education, National Taiwan University of Sport, Taichung 40404, Taiwan; jzlin@ntus.edu.tw

**Keywords:** jump rope, plantar pressure, electromyography

## Abstract

Footwear is among the most important equipment in sports to decrease injuries and enhance performance during exercise. In this study, we investigated differences in lower extremity plantar pressure and muscle activations during jump rope activities. Ten participants performed jump rope under two landing conditions with different footwear. A force platform (AMTI, 1000 Hz), a Novel Pedar-X system (Nove, 100 Hz), and a wireless electromyography (EMG) system (Noraxon, 1500 Hz) were used to measure biomechanical parameters during the jump rope exercise. Vertical ground reaction forces (vGRF), plantar pressure, and lower extremity muscle activations were analyzed. One-leg landing resulted in a significantly greater vGRF and shorter fly time than two-leg landing (*p* < 0.05). A significantly higher peak pressure and lesser toe (LT) area pressure was shown with the jumping shoe (all *p* < 0.05), but lower plantar pressure resulted in the middle foot area (*p* < 0.05). The EMG results of tibialis anterior (TA) were significantly greater with one-leg landing (all *p* < 0.05) during the pre- and background activity (BGA) phases. The results suggest that plantar pressure distribution should be considered when deciding on footwear during jump rope exercises, but care should be taken with regards to recovery after repeated collisions and fatigue. The jumping shoe provides benefits in terms of decreased plantar pressure sustained during jump rope exercises.

## 1. Introduction

Due to the global coronavirus disease 2019 (COVID-19) pandemic, people are continuing to stay at home and take advantage of accessible equipment to maintain physical activities (PA). In 2022, the “home-based exercise” concept arose as an ACSM fitness trend [1], using simple equipment for health promotion or self-training. Under these circumstances, jump rope is a recommended PA, given that it is highly accessible, enjoyable, and affordable for anyone [2,3]. Numerous studies have shown that the jump rope is beneficial to cardiorespiratory health [4], strength [5], agility [6], coordination [7], and bone health [8]. Similar benefits are observed in children [9]. However, a study on home exercise equipment-related injuries indicated that the proportion of injuries caused by jump ropes was 26.3%, with most injuries involving the ankle (79.1%) [10], whereas appropriate shoes may decrease injury risk [11]. Therefore, footwear selection is particularly important in jump rope exercise.

Footwear is the interface between the foot and the ground that protects the plantar, provides shock absorption against repetitive impact, and stabilizes the feet [12]. Footwear is also designed to enhance the performance of movement and foot–ground interaction during exercise [13]. Poorly fitting shoes have a negative effect on muscles and bones and may lead to foot pain [14]. Sports footwear choices should be made on the basis of the type of activity rather than on an individual basis [12]. In the context of exercising, performance, comfort, and injury prevention should be considered in footwear selection [12]. Some characteristics of footwear, such as stability, flexibility, weight, and sole structure, are modified in the name of performance and injury prevention [13]. However, people who participate in exercise are more focused on the protective role of footwear in helping to prevent either acute injury or chronic damage during sporting activities [12,13,14].

The characteristics of jump rope include continuous hopping, wherein the majority of jumping forces are absorbed by the ankle and knee because of the relative rigidity of the lower extremity [15]. Therefore, ankle injury prevention is an important issue in jump rope exercises [10], and appropriate footwear may help to decrease injuries caused by jump rope [16]. Previous studies have reported on vertical ground reaction force (vGRF) [17,18,19,20], plantar pressure [20], joint kinetics [21], kinematics [17,18], and electromyography (EMG) [22,23] as parameters to consider during jump rope exercises. These studies primarily compared jump rope and similar actions, such as hopping [23], and assessed the difference in hardness of the midsoles of different shoes [22]. However, there is a lack of investigation into the effects of different footwear on the biomechanical parameters of injury risk in the jumper. Currently, there is a trend in many sports towards selecting minimalist footwear for performance and training, yet the efficacy of these types of footwear has not been established [24]. Therefore, the purpose of this study was to evaluate the influence of jump-rope- and running-specific footwear on the plantar pressure and lower extremity EMG of one- and two-leg consecutive single movements of jump rope. However, we hypothesized that the distribution and magnitude of lower-limb muscle activations and plantar pressure parameters would be different depending on footwear.

## 2. Materials and Methods

### 2.1. Participants

Ten healthy physical education graduate students (age 23 ± 1.3 years, height 165.6 ± 8.3 cm, body mass 58.6 ± 8.7 kg) were recruited (5 male and 5 female). Participants were selected that had ability to complete at least 140 single under-rope jumps in 1 min. The participants were free of any lower extremity injuries for 6 months prior to the tests. All the participants read and signed informed consent before the experiment. This research was conducted according to the guidelines of the Declaration of Helsinki and approved by the Ethics Committee of the University of Taipei (IRB-2016-021).

### 2.2. Instruments

Force data were collected using a force platform (60 × 90 cm AMTI BP600900-6-2000; Advance Mechanical Technology, INC., Watertown, MA, USA) at a 1000 Hz sampling rate. Plantar pressures were collected using a Novel Pedar-X pressure distribution insole (Novel GmbH, Munich, Germany) at a 100 Hz sampling rate. There were 99 force sensors contained in the insole, with a spatial resolution of approximately 10 mm and a measuring range up to 600 kPa. The system has previously been shown to have high reliability and good validity of plantar pressures [25]. A wireless surface electrode (Noraxon USA Inc., Scottsdale, AZ, USA) was used to record the muscle activations of the vastus medialis (VM), biceps femoris (BF), tibialis anterior (TA), and gastrocnemius muscle (GA) of the dominant leg at a 1500 Hz sampling rate [22].

### 2.3. Procedures

A 5 min dynamic warmup included jump rope practice and leg muscle stretching. Then, a 3 min walk was performed to help accustomate the participants to the backpacks and insoles. The skin was shaved and swabbed with alcohol to decrease EMG artifacts before the electrode piece was attached. Participants were then tested for their maximal voluntary isometric contraction (MVC) on four muscles for the sake of normalization [22]. Each muscle was voluntarily contracted and held with maximal effort for 5 s. Pedar insoles were placed in both shoes, and the insole was connected to the Pedar-X box in a backpack, which was attached to the waist of each participant.

**Figure 1:**
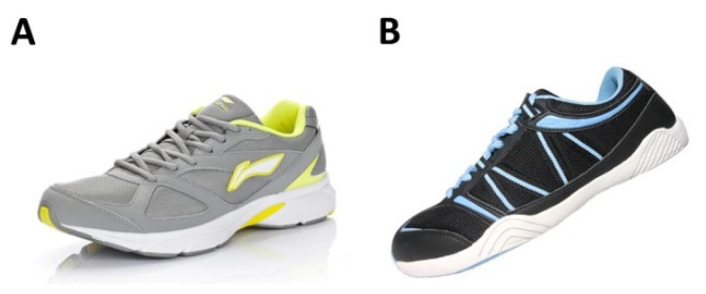
Test footwear: (**A**) running shoe; (**B**) jumping shoe.

Subsequently, the jump rope test was conducted. Each participant was randomly assigned to perform one- and two-leg jump rope trials on a force platform with two testing conditions—running shoe (Li Ning, Beijing, China) and jumping shoe (Jingyuan, Hongkong, China)—at a tempo of 2.2 Hz under metronome guidance. The footwear is shown in Figure 1. The most differentiated of the test footwear was the design of forefoot area; that of the running shoe was upturned, whereas that of the jumping shoe was flat. There were also some differences in the thickness of the midsole; that of the running shoe (forefoot 20 mm, heel 29 mm) was thicker than that of the jumping shoe (forefoot 17.6 mm, heel 26.7 mm). Each trial was undertaken for 30 s to ensure stable jumping was performed [17,22]. Five consecutive cycles of jump rope exercises (ground contact—flight—ground contact) were selected for further analysis from the 30 s of data collected. Participants had a 1 min rest between trials.

### 2.4. Data Reduction and Analysis

**Figure 2:**
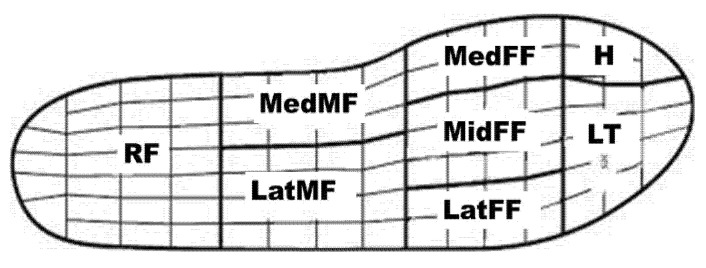
Definition of the plantar pressure regions: H = hallux; LT = lesser toes; MedFF = medial forefoot; MidFF = middle forefoot; LatFF = lateral forefoot; MedMF = medial midfoot; LatMF = lateral midfoot; RF = rearfoot.

Ground contact and flight time were measured by the force platform. Plantar pressure was processed using Pedar Multiproject-ip software (Novel Electronics, New Delhi, India), which divided the foot into 8 regions (Figure 2) for measurement [26]. The discrete plantar pressure measures extracted for statistical analysis were maximum force, peak pressure, average pressure, and force–time integral. The vGRF data were normalized to body weight (BW). The variables of the dominant leg were used for analysis, and this was defined as the segment that would be used to kick a ball [27]. Raw EMG signals were modified by a band-pass filter at 20–400 Hz and full-wave-rectified to denoise and remove artefacts. The root mean square (RMS) algorithm was calculated using a window length of 50. Four EMG phases were analyzed during landings [10,11]. The 100 ms prior to ground contact was defined as the pre-activation (PRE), 30 ms after ground contact was defined as the background activity (BGA), the 30–60 ms after ground contact was defined as a short-latency stretch reflex component (M1) from the spinal reflex, and the 60–90 ms after the ground contact was defined as the long-latency stretch reflex component (M2) by voluntary muscle activity.

### 2.5. Statistical Analysis

SPSS software (IBM SPSS Statistics 21.0, Somers, New York, NY, USA) was used for statistical analysis. Descriptive data are presented as the means (M) and standard deviations (SD) of the parameters. Data homogeneity was tested using Levene’s test, and Shapiro–Wilk tests were conducted to assess the normal distribution. Two-way repeated measures ANOVA was used to determine the differences between landing conditions and footwear during jump rope exercises. Tukey’s honestly significant difference (HSD) test post hoc analysis was then conducted when the level of significance was met; the level of significance for all statistical tests was *p* = 0.05.

## 3. Results

The results of the variations in the main effects and the simple main effects on footwear and landing conditions are shown in Table 1 and Table 2. The force platform results show that the vGRF and peak vGRF were significantly greater in two-leg landing (all *p* < 0.05). No significant difference was found in contact time (*p* > 0.05). There was an interaction found between footwear and landing conditions in terms of flight time. The simple main effects of flight time show that two-leg landing was significantly longer than one-leg landing (Table 1).

The plantar pressure results show that the peak force, peak pressure, average pressure, H area pressure, pressure–time integral, and force–time integral were significantly greater in one-leg landing (all *p* < 0.05). Moreover, significantly higher peak pressure and LT pressure were attained with the jumping shoe (all *p* < 0.05). No significant difference was found in RF and forefoot areas (all *p* > 0.05). There was an interaction between footwear and landing conditions in terms of Lat MF and Med MF pressure. The simple main effects of Lat MF and Med MF pressure were significantly smaller in two-leg landing than in one-leg landing (both *p* < 0.05), and we also found a significantly lower pressure with the jumping shoe (both *p* < 0.05) (Table 2).

**Figure 3:**
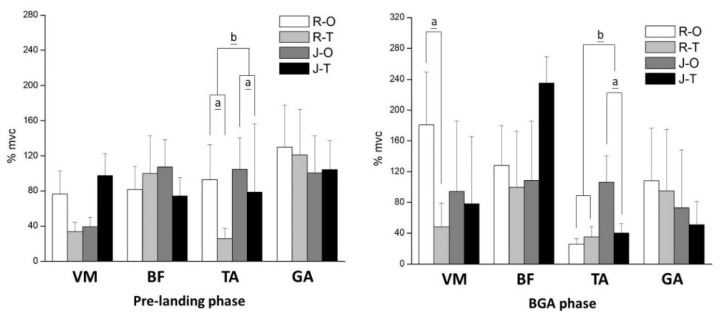

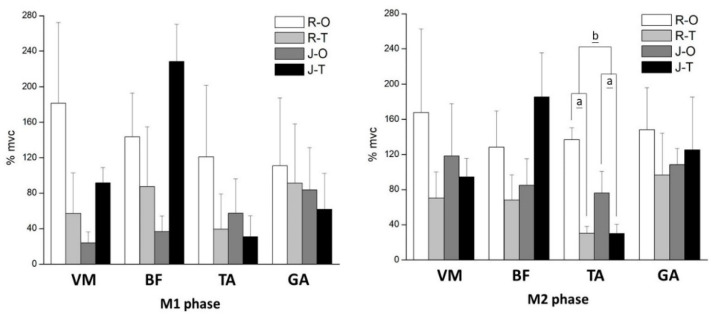
EMG results in different phases. a means significant difference between one- and two-leg landing during jump rope; b means significant difference between running shoe and jumping shoe. R-O = running shoe and one-leg landing; R-T = running shoe and two-leg landing; J-O = jumping shoe and one-leg landing; J-T = jumping shoe and two-leg landing. VM = vastus medialis; BF = biceps femoris; TA = tibialis anterior; GA = gastrocnemius muscle; %mvc = normalized EMG percentage.

The EMG results show that the TA was significantly greater in one-leg landing (all *p* < 0.05) during the pre, BGA, and M2 phases (Figure 3). No significant difference was found in BF and GA (*p* > 0.05). There was an interaction between footwear and landing conditions in terms of VM and TA during the pre, BGA, and M2 phases. The simple main effects of TA in two-leg landing were significantly smaller than in one-leg landing (both *p* < 0.05), and we also found significantly fewer muscle activations with the jumping shoe (both *p* < 0.05).

## 4. Discussion

The purpose of this study was to evaluate the influence of different footwear on the plantar pressure and lower extremity EMG during jump rope exercise. The results demonstrate that there were greater differences in vGRF and plantar pressure variables with different landing conditions with different footwear. The plantar pressures were greater when wearing running shoes in peak pressure, Lat MF, Med MF, and LT areas. Additionally, one-leg landing showed higher muscle activation in VM. The TA shows smaller muscle activation with two-leg landing and wearing jumping shoes. These findings may offer an important implication for those planning integrate jump rope into their training programs.

A larger landing force was measured with the force platform than with Pedar system, which is consistent with previous research [28]. In this study, the force platform data reflect the total ground reaction force produced during the jump rope activity (both land in a force platform), which acts on the whole body, whereas the data from the Pedar sensors were restricted to the contact areas of the sensors, which measured a smaller force value than the platform [29]. Second, the difference between the two landing conditions affects the ground contact area (one-leg and two-leg landing), which may influence the force value calculated by the Pedar sensors. Therefore, in the present research, the term “force” and “vGRF” were used to represent landing force from the two devices (Table 1). Previous research indicated that the jump rope technique may have a smaller ground contact area with increased jumping frequency [21], which manifests as stiffness of jump movement [23]. This may influence the force measurement from the Pedar insole but not the force platform. However, previous research has shown a good accuracy and reliability between devices, be it the force plate or the insole [28]. However, the force data of the Pedar insole was more implicated in the present research to reflect one- and two-legged jump rope, as the insole pressure measurements have a better applicability compared with those of the force platform.

A significant difference in peak plantar pressure was found, with the main difference coming from the forefoot (H and LT areas) and midfoot (Lat MF and Med MF areas), which may have been affected by the different forefoot design and midsole thickness of the footwear. Previous research has indicated that the peak pressure mainly occurs in the metatarsal heads and thumbs during jump rope exercises [20], and research using finite element analysis has suggested that except for in the customized model, arch design was most influential in reducing plantar pressure [30]. In the current result, however, there was an inevitable difference between one- and two-leg jump rope, which are totally different skills [31]. The jumping shoe shows a higher plantar pressure in the lesser forefoot but a smaller pressure in the midfoot, which might be caused by differences in shoe structure design, such as the flat structure in the forefoot and thinner midsole. The higher pressures in the midfoot may increase the fascia stress [32] and accelerate fatigue [33]. From this perspective, the jumping shoe may be suitable for long-term jumping exercise.

In the current study, the TA was significantly greater in the pre and BGA phases when wearing the jumping shoe and performing one-leg landing, but higher activation occurred in the M2 phase when wearing the running shoe. Generally, the pre-activity and BGA phases are driven by signals from the central nervous system, which reflect the reflex sensitivity by modulating the excitability of the motor neuron pool [23], and the M2 phase is considered to be voluntarily activated by a supraspinal command, which is classified as a long-latency reflex [34]. The mechanisms of these muscle activities adjust joint stiffness to predict the landing impact; specifically, the processes of preparatory (pre-phase) and reflexive (BGA-M2 phase) muscle activation contribute to functional joint stability, which is also considered a protective mechanism that decreases lower extremity stiffness during impact [35]. Previous research has suggested that when performing repeated strength-shortening cycle movements, higher lower-body stiffness appeared to be advantageous [36]. Thus, the higher level of TA muscle activation in the current study may reflect greater lower extremity stiffness with one-leg jumping and when wearing jumping shoes during jump rope exercises. Although lower extremity stiffness was not measured, we inferred the possible causes of the higher TA activation from the combined plantar pressure results. However, we do not have enough evidence to prove the inference, which necessitates a more comprehensive investigation in the future.

Some limitations of this study should be mentioned. The sample size was small, and the technique differences may be affected by skill level [17,18]. Lower extremity stiffness was not measured in this study, which is something future investigations should address. Additionally, kinematics data must be investigated, which will help us better understand the influence of shoe differences during jump rope exercises.

## 5. Conclusions

The present study investigated the effects of different footwear and landing conditions on plantar pressure, vertical ground reaction force, and lower extremity muscle activation during jump rope exercises. The footwear structural design may influence the plantar pressure in the midfoot area, and the movements of the jump rope activity were mainly focused in the forefoot area. Higher TA muscle activation was observed with jumping shoes than with running shoes during jump rope exercises, and this might reveal better rebound of the jumping shoe. Although the difference between running and jumping shoes may be minor, these may also have been concealed by jump rope skill level. The results suggest that jumping shoes cause lower plantar pressure, which may be suitable for prolonged jump rope exercises. It is important to consider footwear function and design when participating in jump rope exercises.

## Figures and Tables

**Table 1:** Descriptive statistics (Mean ± SD) and statistical results of force platform variables during the jump rope task.

Footwear	Running Shoe		Jumping Shoe		*p*-Values		
Variables	One-Leg Jump	Two-Leg Jump	One-Leg Jump	Two-Leg Jump	Interaction	Footwear	Condition
vGRF (N) ^c^	1829.04 ± 320.14	2511.32 ± 516.40	2045.33 ± 638.47	2494.61 ± 588.51	*p* = 0.220	*p* = 0.275	*p* < 0.001
Peak vGRF (N) ^c^	1936.53 ± 351.39	2610.48 ± 529.64	2146.99 ± 628.34	2574.54 ± 591.77	*p* = 0.209	*p* = 0.345	*p* < 0.001
vGRF (BW) ^c^	3.07 ± 0.36	4.21 ± 0.53	3.39 ± 0.64	4.17 ± 0.60	*p* = 0.173	*p* = 0.283	*p* < 0.001
Peak vGRF (BW) ^c^	3.27 ± 0.43	4.38 ± 0.55	3.57 ± 0.62	4.30 ± 0.59	*p* = 0.175	*p* = 0.390	*p* < 0.001
Contact time (ms)	289.06 ± 104.36	248.38 ± 71.33	297.54 ± 88.82	249.98 ± 74.81	*p* = 0.670	*p* = 0.498	*p* = 0.054
Flight time (ms) ^a,c^	235.54 ± 57.39	297.8 ± 93.80	238 ± 59.65	283.56 ±9 3.15	*p* = 0.040	*p* = 0.274	*p* = 0.002

^a^ means significant interaction of footwear and landing condition. ^c^ means significant difference between one- and two-leg landing during jump rope.

**Table 2:** Descriptive statistics (Mean ± SD) and statistical results of plantar pressure variables during the jump rope task.

Variables	Running Shoe		Jumping Shoe		*p*-Values		
	One-Leg Jump	Two-Leg Jump	One-Leg Jump	Two-Leg Jump	Interaction	Footwear	Condition
Peak force (N) ^c^	1579.83 ± 392.28	987.60 ± 227.21	1552.26 ± 330.29	1097.13 ± 272.73	*p* = 0.150	*p* = 0.195	*p* < 0.001
Peak force (BW) ^c^	2.66 ± 0.52	1.71 ± 0.28	2.61 ± 0.42	1.84 ± 0.31	*p* = 0.189	*p* = 0.376	*p* < 0.001
Peak pressure (kpa) ^b,c^	438.57 ± 122.37	324.76 ± 66.60	469.34 ± 110.76	391.90 ± 148.69	*p* = 0.453	*p* = 0.044	*p* = 0.005
Average pressure (kpa) ^c^	139.071 ± 23.82	115.38 ± 23.44	149.998 ± 32.43	128.49 ± 39.88	*p* = 0.841	*p* = 0.073	*p* = 0.006
RF (kpa)	63.13 ± 29.20	30.88 ± 26.21	40.22 ± 59.37	32.39 ± 66.63	*p* = 0.456	*p* = 0.385	*p* = 0.223
Lat MF (kpa) ^a,b,c^	183.79 ± 60.33	89.81 ± 59.57	114.52 ± 84.66	103.34 ±5 8.51	*p* = 0.036	*p* = 0.001	*p* = 0.001
Med MF (kpa) ^a,b,c^	125.55 ± 32.13	43.09 ± 37.73	58.52 ± 37.09	28.28 ± 30.38	*p* = 0.007	*p* < 0.000	*p* < 0.001
Med FF (kpa)	229.77 ± 81.09	272.70 ± 174.04	296.42 ± 216.99	283.61 ± 158.99	*p* = 0.481	*p* = 0.290	*p* = 0.533
Mid FF (kpa)	364.83 ± 68.14	412.97 ± 178.76	453.78 ± 127.85	463.62 ± 148.05	*p* = 0.526	*p* = 0.107	*p* = 0.430
Lat FF (kpa)	157.08 ± 7.74	185.47 ± 67.44	198.05 ± 42.33	217.29 ± 101.06	*p* = 0.711	*p* = 0.207	*p* = 0.059
H (kpa) ^c^	136.56 ± 33.73	196.06 ± 73.11	144.72 ± 43.23	179.48 ± 24.58	*p* = 0.418	*p* = 0.798	*p* = 0.002
LT (kpa) ^b^	131.57 ± 27.42	219.27 ± 99.52	179.31 ± 66.88	186.31 ± 42.29	*p* = 0.291	*p* = 0.018	*p* = 0.709
P × T (kpa∙s) ^c^	5069.13 ± 1747.14	3562.34 ± 1396.92	4651.14 ± 1733.35	4393.57 ± 1116.79	*p* = 0.072	*p* = 0.714	*p* = 0.039
F × T (N∙s) ^c^	14,701.38 ± 5390.83	8855.83 ± 3120.25	13,049.98 ± 4451.39	8517.45 ± 4085.79	*p* = 0.519	*p* = 0.242	*p* < 0.001

^a^ means significant interaction between footwear and landing condition. ^b^ means significant difference between running shoe and jumping shoe. ^c^ means significant difference between one- and two-leg landing during jump rope. RF = rearfoot; LatMF = lateral midfoot; MedMF = medial midfoot; MedFF = medial forefoot; MidFF = middle forefoot; LatFF = lateral forefoot; H = hallux; LT = lesser toes; P = pressure; T = time; F = force.

## Data Availability

Data is contained within the article.

## References

[1] Thompson W.R. (2021). Worldwide survey of fitness trends for 2022. ACSMs Health Fit. J..

[2] Ha A.S., Lonsdale C., Ng J.Y.Y., Lubans D.R. (2014). A School-Based Rope Skipping Intervention for Adolescentsin Hong Kong: Protocol of a Matched-Pair Cluster Randomized Controlled Trial. BMC Public Health.

[3] Ha A.S., Burnett A., Sum R., Medic N., Ng J.Y.Y. (2015). Outcomes of the Rope Skipping “STAR” Programme for School children. J. Hum. Kinet..

[4] Quirk J.E., Sinning W.E. (1982). Anaerobic and aerobic responses of males and females to rope skipping. Med. Sci. Sports Exerc..

[5] Duzgun I., Baltaci G., Colakoglu F., Tunay V.B., Ozer D. (2010). The effects of jump-rope training on shoulder isokinetic strength in adolescent volleyball players. J. Sport Rehabil..

[6] Miyaguchi K., Demura S., Omoya M. (2015). Relationship between jump rope double unders and sprint performance in elementary schoolchildren. J. Strength Cond. Res..

[7] Ozer D., Duzgun I., Baltaci G., Karacan S., Colakoglu F. (2011). The effects of rope or weighted rope jump training on strength, coordination and proprioception in adolescent female volleyball players. J. Sport Med. Phys. Fit..

[8] Pettersson U., Nordstro P., Alfredson H. (2000). Effect of high impact activity on bone mass and size in adolescent females: A comparative study between two different types of sports. Calcif. Tissue Int..

[9] Eler N., Acar H. (2018). The Effects of the Rope Jump Training Program in Physical Education Lessons on Strength, Speed and VO2Max in Children. Univers. J. Educ. Res..

[10] Martinez A., Snyder A.J., Smith G.A. (2011). Home Exercise Equipment-Related Injuries Among Children in the United States. Clin. Pediatr..

[11] Theisen D., Malisoux L., Gette P., Nührenbörger C., Urhausen A. (2016). Footwear and running-related injuries: Running on faith?. Sports Orthop. Traumatol..

[12] Prado M.P., Saito G.H., Rocha Piedade S., Imhoff A., Clatworthy M., Cohen M., Espregueira-Mendes J. (2019). Sports Footwear: Problems and Advances. The Sports Medicine Physician.

[13] Lake M.J. (2000). Determining the protective function of sports footwear. Ergonomics.

[14] Chiou W., Chiu H., Chao A., Wang M., Chen Y. (2015). The influence of body mass on the foot dimensions during pregnancy. Appl. Ergon..

[15] Lamontagne M., Kennedy M.J. (2013). The biomechanics of vertical hopping: A review. Res. Sports Med..

[16] Shorten M.R. (1993). The energetics of running and running shoes. J. Biomech..

[17] Jang K.H., Son M.J., Kim D.Y., Lee M.G., Kim Y.K., Kim J.H., Youm C.H. (2017). Effects of skill level and feet width on kinematic and kinetic variables during jump rope single under. Korean J. Sport Biomech..

[18] Kim D.Y., Jang K.H., Lee M.G., Son M.J., Kim Y.K., Kim J.H., Youm C.H. (2017). Analysis of kinematics and kinetics according to skill level and sex in double-under jump rope technique. Korean J. Sport Biomech..

[19] Pittenger V.M., McCaw S.T., Thomas D.O. (2002). Vertical ground reaction forces of children during one- and two-leg rope jumping. Res. Q. Exerc. Sport.

[20] Shek M.C., Fong D.T.P., Hong Y. Ground reaction forces and plantar kinetics of rope skipping in different sports shoes–A pilot study. Proceedings of the 23rd International Symposium on Biomechanics in Sports.

[21] Bruce O.L., Ramsay M., Kennedy G., Edwards W.B. (2020). Lower-limb joint kinetics in jump rope skills performed by competitive athletes. Sports Biomech..

[22] Yu H.-B., Tai W.-H., Li J., Zhang R., Hao W.-Y., Lin J.-Z. (2021). Effects of Shoe Midsole Hardness on Lower Extremity Biomechanics during Jump Rope in Healthy Males. Healthcare.

[23] Hobara H., Inoue K., Muraoka T., Omuro K., Sakamoto M., Kanosue K. (2010). Leg stiffness adjustment for a range of hopping frequencies in humans. J. Biomech..

[24] Sinclair J., Chockalingam N., Naemi R., Vincent H. (2014). The effects of sport-specific and minimalist footwear on the kinetics and kinematics of three netball-specific movements. Footwear Sci..

[25] Putti A.B., Arnold G.P., Cochrane L., Abboud R.J. (2007). The Pedar in-shoe system: Repeatability and normal pressure values. Gait Posture.

[26] Queen R.M., Mall N.A., Nunley J.A., Chuckpaiwong B. (2009). Differences in plantar loading between flat and normal feet during different athletic tasks. Gait Posture.

[27] Van Melick N., Meddeler B.M., Hoogeboom T.J., Nijhuis-van der Sanden M.W.G., van Cingel R.E.H. (2017). How to determine leg dominance: The agreement between self-reported and observed performance in healthy adults. PLoS ONE.

[28] Barnett S., Cunningham J.L., West S. (2001). A comparison of vertical force and temporal parameters produced by an in-shoe pressure measuring system and a force platform. Clin. Biomech..

[29] Weizman Y., Tan A.M., Fuss F.K. (2019). Benchmarking study of the forces and centre of pressure derived from a novel smart-insole against an existing pressure measuring insole and force plate. Measurement.

[30] Cheung J.T., Zhang M. (2008). Parametric design of pressure-relieving foot orthosis using statistics-based finite element method. Med. Eng. Phys..

[31] Yu H.B., Monchai C., Tsai Y.S. (2018). Effects of athletic footwear on plantar force during rope skipping. Int. J. Exp. Comput. Biomech..

[32] Hsu Y.C., Gung Y.W., Shih S.L., Feng C.K., Wei S.H., Yu C.H., Chen C.S. (2008). Using an Optimization Approach to Design an Insole for Lowering Plantar Fascia Stress—A Finite Element Study. Ann. Biomed. Eng..

[33] Weist R., Eils E., Rosenbaum D. (2004). The influence of muscle fatigue on electromyogram and plantar pressure patterns as an explanation for the incidence of metatarsal stress fractures. Am. J. Sports Med..

[34] Hobara H., Kanosue K., Suzuki S. (2007). Changes in muscle activity with increase in leg stiffness during hopping. Neurosci. Lett..

[35] Pithioux M., Chavet P., St-Onge N., Nicol C. (2005). Influence of muscle preactivation of the lower limb on impact dynamics in the case of frontal collision. Int. J. Crashworthiness.

[36] Pruyn E.C., Watsford M., Murphy A. (2014). The relationship between lower-body stiffness and dynamic performance. Appl. Physiol. Nutr. Metab..

